# A short-term cooling of root-zone temperature increases bioactive compounds in baby leaf *Amaranthus tricolor* L.

**DOI:** 10.3389/fpls.2022.944716

**Published:** 2022-07-15

**Authors:** Takon Wittayathanarattana, Praderm Wanichananan, Kanyaratt Supaibulwatana, Eiji Goto

**Affiliations:** ^1^Graduate School of Horticulture, Chiba University, Chiba, Japan; ^2^Department of Biotechnology, Faculty of Science, Mahidol University, Bangkok, Thailand; ^3^National Science and Technology Development Agency, Thailand Science Park, National Center for Genetic Engineering and Biotechnology, Pathum Thani, Thailand; ^4^Plant Molecular Science Center, Chiba University, Chiba, Japan

**Keywords:** abiotic stress, anthocyanin, antioxidant capacity, ascorbic acid, baby greens, betalain, flavonoids, seedling

## Abstract

Leafy vegetables that are offered as seedling leaves with petioles are referred to as baby leaf vegetables. One of the most nutritious baby leaves, amaranth (*Amaranthus tricolor* L.), contains several bioactive compounds and nutrients. Here, we investigated the growth and quality of baby leaf amaranth using a variety of short-term cooling root-zone temperatures (RZT; 5, 10, 15, and 20°C), periods (1, 3, 5, and 7 days), and combinations thereof. We observed that exposing amaranth seedlings to RZT treatments at 5 and 10°C for 1–3 days increased the antioxidant capacity and the concentrations of bioactive compounds, such as betalain, anthocyanin, phenolic, flavonoid, and ascorbic acid; however, extending the treatment period to 7 days decreased them and adversely affected growth. For RZT treatments at 20°C, leaf photosynthetic pigments, bioactive compounds, nutrients, and antioxidant capacity increased gradually as the treatment period was extended to 7 days. The integration of RZTs at 5 and 10°C for one day preceded or followed by an RZT treatment at 20°C for 2 days had varied effects on the growth and quality of amaranth leaves. After one day of RZT treatment at 5°C followed by 2 days of RZT treatment at 20°C, the highest concentrations of bioactive compounds, nutrients, and antioxidant capacity were 1.4–3.0, 1.7, and 1.7 times higher, respectively, than those of the control, and growth was not impaired. The short-term cooling RZT treatments under controlled environments were demonstrated to be adequate conditions for the improvement of target bioactive compounds in amaranth baby leaf without causing leaf abnormality or growth impairment.

## Introduction

Consumption of food with health benefits, often called functional foods, is increasing. Functional foods could prevent lifestyle diseases, such as cancer, atherosclerosis, and Alzheimer’s disease due to their bioactive compounds (Lee et al., 2009; Albuquerque et al., 2021). Vegetables represent affordable daily functional foods that contain various bioactive compounds, such as flavonoids, phenylpropanoids, and carotenoids.

Plants have been treated with biotic and abiotic stress to enhance the concentrations of their bioactive compounds. Root-zone temperature (RZT) is one of the abiotic factors that could enhance the contents of plant bioactive compounds (Calleja-Cabrera et al., 2020). The impact of RZT on growth and bioactive compound contents in relation to nutrition and water uptake functions in plants has been investigated (Qiuyan et al., 2012). Low or high RZT induces water stress in plants by altering the fluidity of the root membrane and the activity of aquaporin, a protein involved in water absorption (Carvajal et al., 1996; Maurel et al., 2015). In addition, calcium channels and lipid signaling are triggered as a consequence of such alterations, hence affecting intracellular calcium ions (Mittler et al., 2012). Subsequently, the calcium ion stimulates the activity of various temperature-sensitive elements, including calcium-dependent protein kinases and heat shock proteins. Additionally, reactive oxygen species (ROS), unstable oxidative products, are accumulated when certain thermal responsive mechanisms are altered (Raja et al., 2017). Numerous bioactive compounds that serve as ROS neutralizers, especially flavonoids and ascorbic acid, are synthesized and exploited to minimize ROS damage (Escobar-Bravo et al., 2017; Rácz and Hideg, 2021). Signal molecules are delivered from the roots to the shoots to trigger a bioactive compound synthesis pathway. However, when the ROS generated by low or high RZT exceed a threshold, cell death occurs (Mackerness, 2000). Moreover, a signal molecule is delivered to the shoots, signaling shoot-to-root nutrition transport, resulting in nutrient and biomass losses from the shoots (Heckathorn et al., 2013). Therefore, RZT treatment not only affects root physiology and activity but also shoot growth.

Root-zone temperature treatment is suitable for application in hydroponic systems. It facilitates a consistent nutrient solution temperature under controlled environments, such as in a plant factory with artificial light or a vertical farm. Ogawa et al. (2018) demonstrated that a 6-day RZT treatment at 10°C enhanced rosmarinic acid and luteolin concentrations in red perilla while decreasing shoot fresh weight. Cucumber plants were exposed to low RZT (10°C) for 4 weeks decreased leaf fresh weight and mineral content in addition to increasing leaf soluble sugar concentration (Chung et al., 2002). Similarly, a short-term RZT treatment (7 days) at 10–15°C enhanced total phenolic, anthocyanin, sugar, and antioxidant enzyme concentrations in red leaf lettuce, while decreasing fresh weight (Sakamoto and Suzuki, 2015). Furthermore, a high-temperature RZT treatment (30°C) increased the concentrations of bioactive compounds but decreased their antioxidant activity and appearance quality. Nguyen et al. (2020) applied RZT treatments to coriander for 3 and 6 days at 15, 25, 30, and 35°C. After 6 days, coriander treated with 15 or 35°C yielded relatively high concentrations of bioactive compounds, including ascorbic acid, chlorogenic acid, and carotenoids, while the highest biomass was observed in the treatments exposed to 30°C. The coriander treated for 3 days at 15°C exhibited decreased total phenolic contents, and no significant difference in fresh weight, when compared with that in the control group. The results of the study indicated that specific RZT levels and exposure periods influence the contents bioactive compounds differently.

Although high RZT treatment may enhance the contents of bioactive compounds in plants, it could have adverse effects on the appearance and shelf life of vegetable products due to increased respiration rates (Chun and Takakura, 1994; Falah et al., 2010; Masarirambi et al., 2018). Consequently, low RZT levels increase bioactive compounds in plants but decrease the water and minerals contents of the final product (Calatayud et al., 2008; Nxawe et al., 2009). Overall, RZT treatments at low and high levels influence bioactive compound characteristics, yield, and plant appearance. Consequently, low or high RZT could be employed considering the intended purpose of the product. Furthermore, rational RZT management could increase the contents of bioactive compounds in plants without adversely affecting growth and appearance.

*Baby leaf* vegetables, also known as baby greens, are commercial terms for leafy vegetables sold as seedling leaves with petioles. The leaf length, leaf area, color, texture, and flavor of baby leaf vegetables and the concentrations of certain bioactive compounds, such as ascorbic acid, anthocyanin, and β-carotene, are major quality criteria (Takahama et al., 2019). A seedling stage plant, on average, contains more bioactive compounds, nutrients, and minerals than a mature plant (Paradiso et al., 2018; Rakpenthai et al., 2019; Le et al., 2020).

Amaranth (*Amaranthus tricolor* L.) is a nutritious leafy vegetable with numerous and abundant bioactive compounds, such as ascorbic acid, betalain, β-carotene, phenolics, and flavonoids (Asao and Watanabe, 2010; Sarker and Oba, 2018; Sarker et al., 2020). Although amaranth baby leaf products are available on the market, no short-term treatment for manipulating the aerial or root-zone environment has been developed to enhance the contents of bioactive compounds in the leaves without causing leaf abnormality.

The aim of the present study was to establish a short-term cooling RZT under controlled environments that could be employed to improve bioactive compound accumulation in baby leaf amaranth without inducing abnormal appearance. RZT level, treatment period, and RZT combinations with different introduction times were investigated.

## Materials and methods

### Plant material and cultivation condition

The experiments were conducted at Chiba University, Japan, in a closed plant production system equipped with a multi-layer cultivation shelf. *A. tricolor* L. (red amaranth) seeds were purchased from Nakahara Seed Product Co., Ltd. (Fukuoka, Japan). The cultivation procedure diagram is illustrated in Supplementary Figure S1. Two hundred seeds were germinated in the dark at 20°C. Ninety-six germinated seeds were sown on a polyurethane sponge (M urethane; M Hydroponics Laboratory Co. Inc., Aichi, Japan) which was placed in a 1.2-L polypropylene cultivation container. Once pairs of true leaves were visible, 56 uniform seedlings were transplanted to an 8-L cultivation container. A half-strength Otsuka A formulation (OAT house A treatment; OAT Agrio Co. Ltd., Tokyo, Japan) with an electrical conductivity of 0.10–0.12 S m^−1^ and pH of 6.3–6.5 was used as the nutrient solution and seedlings were irradiated with white LED lamps (LD40S-N/19/21; Panasonic Corporation, Osaka, Japan). The environmental condition until 26 days after sowing (DAS) is described in Table 1.

**Table 1:** Environmental conditions for *Amaranthus tricolor* L. seedling stage and test period.

Environmental factor	Setting value
PPFD (μmol m^−2^ s^−1^) for seedling stage	200
PPFD (μmol m^−2^ s^−1^) for test period	300
Light period (h)	16
Air temperature (light/dark, °C)	25/20
Nutrient solution temperature (°C)	Not controlled
Relative humidity (%)	70
CO_2_ concentration (μmol mol^−1^)	1,000

PPFD, photosynthetic photon flux density.

### Root-zone temperature treatments

Amaranth seedlings with four true leaves, approximately 27 DAS, were exposed to RZT treatments. Six seedlings selected from the average height seedlings grown in the 8-L container were transplanted into a 6-L hydroponic container (33 cm × 18 cm × 15 cm) for the treatment (Supplementary Figure S1). The RZT was controlled using a handy cooler (TRL-107NHF, Thomas Kagaku Co., Ltd., Tokyo, Japan) with a temperature control device (Supplementary Figures S2, S3). A 1.5-cm-diameter cooling coil and thermocouple connected to a temperature control device were installed in the treatment container. Aeration was performed using air pumps and air stones to ensure adequate supply of air to the roots and circulation of the nutrient solution in each container. The environmental conditions during the test period are described in Table 1. Seedlings in all treatments were exposed to similar aerial conditions.

Experiment 1 was carried out to determine the optimal RZT and treatment period. The seedlings were subjected to four RZT treatments for 1, 3, 5, and 7 days, including 5, 10, 15, and 20°C. Four seedlings were harvested after 1, 3, 5, and 7 days of treatment.

Experiment 2 was carried out to establish RZT integration regimes. The results from Experiment 1 demonstrated that RZT at 5 and 10°C for 1 day increased concentrations of targeted bioactive compounds. Furthermore, RZT at 20°C for one or 3 days increased or maintained photosynthetic pigment concentrations. Thus, we hypothesized that integrating RZT at 5 or 10°C for 1 day and 20°C for 2 days could synergistically enhance and maintain bioactive compounds of baby leaf amaranth. The seedlings were subjected to four RZT treatments (20 T5, 20 T10, 5 T20, and 10 T20°C). Table 2 and Supplementary Figure S4 show the RZT treatments and periods. Three days after treatment, four seedlings were harvested.

**Table 2:** Short-term root-zone temperature (RZT) conditions for the treatment of amaranth seedlings. The seedlings were irradiated under the same photosynthetic photon flux density of 300  μmol m^−2^ s^−1^ for 3 days (Experiment 2).

Treatment	RZT treatment (day/night, °C)		
	Day-1	Day-2	Day-3
20 T5	20/20	20/20	5/5
20 T10	20/20	20/20	10/10
5 T20	5/5	20/20	20/20
10 T20	10/10	20/20	20/20

Seedlings in all treatments were exposed to the same aerial conditions. The RZT of the control was maintained almost the same as the air temperature during a 25/20°C temperature cycle (day/night). The experiments were performed once.

Air temperatures in the canopy, cultivation shelf, and under cultivation foam were determined using a humidity and temperature recorder (TR-72wf, T&D Co., Ltd., Matsumoto, Japan; Supplementary Table S1). An infrared thermometer (830-T2, Testo, Inc.) was used to determine the surface temperature of the leaf and expanded polystyrene cultivation foam, whose emissivity was adjusted to 0.98 and 0.95, respectively (Chen, 2015; Krause and Nowoświat, 2019). The temperature of the nutrient solution was determined at different areas using thermocouples from the temperature control device on a handy cooler.

### Yield, leaf water content, and leaf morphology

Four or five younger amaranth leaves 4.0–5.0 cm in length were harvested from four uniform seedlings, weighed, and subsequently freeze-dried (FDU-1110; Tokyo Rikakikai Co. Ltd., Tokyo, Japan) at −80°C for 24 h before being stored at −30°C for further analyses. Fresh (FW) and dry (DW) leaf weights were determined using a digital balance. The leaf water content (LWC) was calculated as previously described (Garnier and Laurent, 1994). Leaf morphology was visually recorded using a mobile camera.

### Biochemicals determination

Sample preparation for biochemical determination was performed according to Wittayathanarattana et al. (2022). The leaves of each seedling were cold ground before being biochemically analyzed.

#### Chlorophyll and carotenoid concentrations

Powdered leaf samples (5 mg) were mixed with 1 ml of cold 80% acetone (v/v) and then homogenized using a 40 W ultrasonic machine. Samples were incubated overnight at 4°C and subsequently centrifuged (MX-307, TOMY Seiko Co., Ltd., Japan). The absorbance of the supernatants at 470, 480, 510, 645, and 663 nm was measured using a spectrophotometer (V-750; JASCO Corp., Japan). Total chlorophyll and carotenoid concentrations (conc.) were calculated using equations previously described by Sarker and Oba (2020).

#### Betalain concentration

Freeze-dried plant tissue samples (5 mg) were extracted with 1 ml of cold 80% methanol (v/v) containing 50 mM ascorbic acid. Samples were homogenized, then incubated overnight at 4°C and subsequently centrifuged. The absorbance of the β-cyanin and β-xanthin pigments were measured at 540 and 475 nm, respectively, using the spectrophotometer. Data were calculated as mg of betanin equivalent g^−1^ DW for β-cyanin and mg of indicaxanthin equivalent g^−1^ DW for β-xanthin. The concentrations were calculated by combining the β-cyanin and β-xanthin concentrations according to Sarker and Oba (2020).

#### Anthocyanin concentration

Anthocyanin determination was performed using a method modified from Mancinelli and Schwartz (1984). Powdered samples (5 mg) were mixed with 0.5 ml of chloroform, homogenized, and thereafter, centrifuged. Residues were collected and mixed with 1 ml of a 1% HCl-methanol (v/v) solution and subsequently centrifuged. The absorbance of supernatant was measured at 530 and 657 nm using the spectrophotometer. An appropriate dilution of cyanidin-3-glucoside (C3G) was used as the standard. The anthocyanin concentration was calculated using the equation described by Stanciu et al. (2009).

#### Total phenolic and flavonoid concentrations

Powdered leaf samples (5 mg) were mixed with 1 ml of cold 90% methanol (v/v), obtaining methanolic extract. The total phenolic concentration determination was performed as described by Jiménez-Aguilar and Grusak (2017). The extracts were mixed with 0.2 N of Folin-Ciocalteu reagent and 1 mol L^−1^ Na_2_CO_3_ at a ratio of 1:5:4. The absorbance of the solutions was measured at 760 nm using the spectrophotometer. Appropriate dilutions of gallic acid were used to plot a standard curve. Total phenolic concentrations were expressed as gallic acid equivalents (mg of GAE g^−1^ DW). Total flavonoid concentrations were determined based on the aluminum chloride reaction principle, as described in Sarker and Oba (2020). The extract was mixed with 5% NaNO_2_ (w/v), 10% aluminum chloride (w/v) solution, and 1 mol L^−1^ NaOH at a ratio of 5:1.5:2:1.5. Absorbance was measured at 410 nm using the spectrophotometer. An appropriate dilution of rutin was used to create a standard curve. The total flavonoid concentrations were expressed as rutin equivalents (mg of RTE g^−1^ DW).

#### Total antioxidant capacity

An appropriate dilution of methanolic extract was mixed with 2,2-azino-bis (3-ethylbenzothiazoline-6-sulfonic acid; ABTS; Sigma-Aldrich, St. Louis, MO, United States of America) according to the method described by Miller and Rice-Evans (1996). The reduction in absorbance at 740 nm within 1 min was measured using the spectrophotometer. An appropriate dilution of Trolox was used to plot a standard curve. Total antioxidant capacity was expressed as Trolox equivalent antioxidant capacity (mM g^−1^ DW).

#### Ascorbic acid concentration

Powdered leaf samples (10 mg) were combined with 1 ml of 5% meta-phosphoric acid (w/v), then mixed well by vortexing for 1 min and centrifuged. Ascorbic acid determination was carried out using Reflectoquant^®^ ascorbic acid test strips (Merck, Germany) and a reflectometer (RQflex^®^, Merck). Ascorbic acid concentration was expressed as mg g^−1^ DW.

### Statistical analysis

Data were processed using IBM SPSS Statistics 24 (IBM Corp., Armonk, NY, United States of America). Tukey’s honestly significant difference (Tukey’s HSD) test was used to compare the means of the measured parameters among the treatments.

## Results

### The optimization of RZT and treatment period

**Figure 1:**
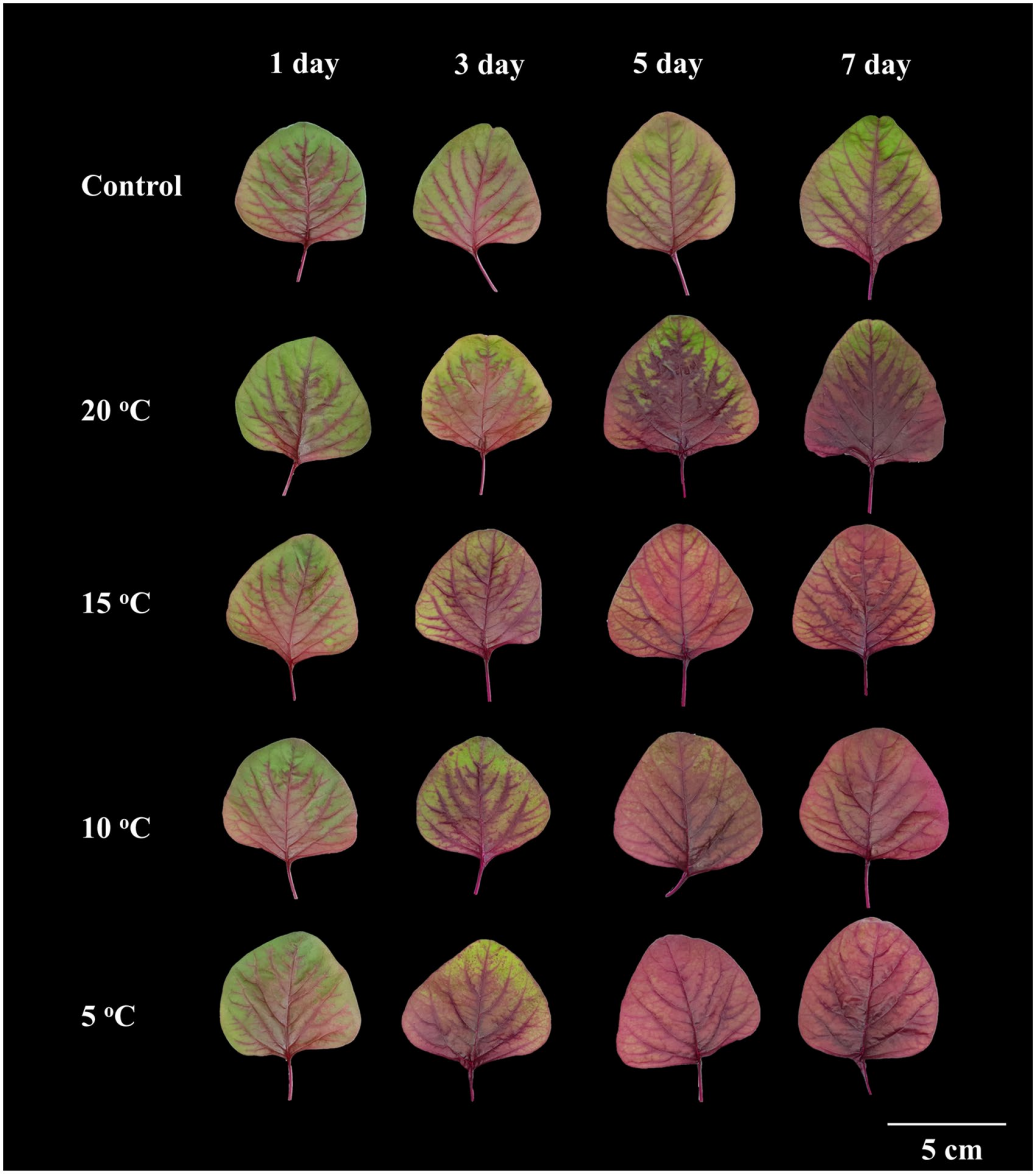
Morphology of amaranth leaves treated with different root-zone temperatures (RZT) for different periods (Experiment 1). The RZT at day/night-time: 5/5, 10/10, 15/15, and 20/20°C are indicated as: 5, 10, 15, and 20°C.

**Figure 2:**
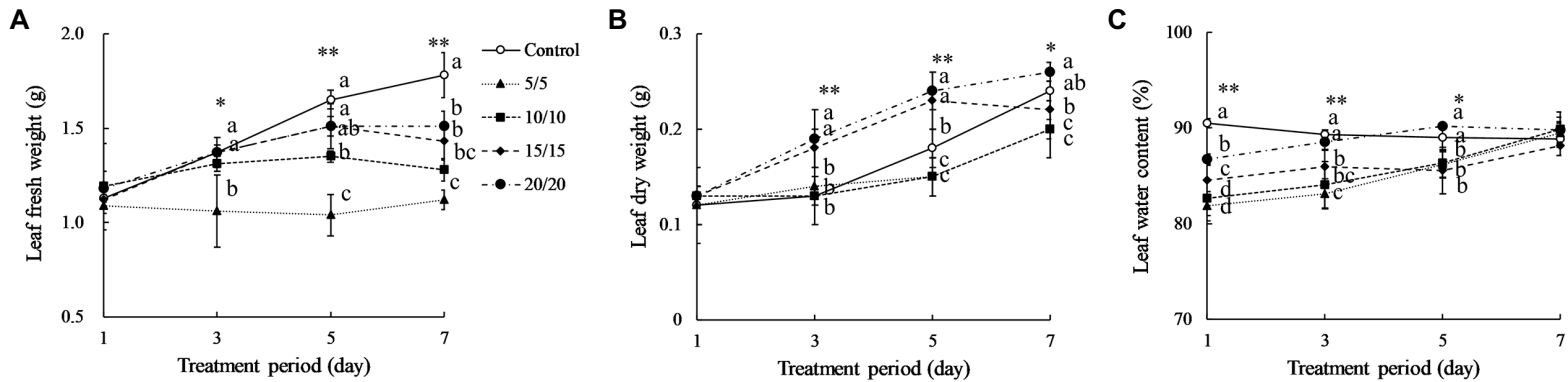
Amaranth leaf fresh weight **(A)**, dry weight **(B)**, and leaf water content **(C)** after treatment with different short-term RZTs for different periods (Experiment 1). Vertical bars indicate the standard error (*n* = 4). Means were compared using Tukey’s honestly significant difference (HSD) at a significance level at ^*^p  <  0.05 and ^**^p  <  0.01.

Amaranth seedlings developed differently under various RTZs. At day 7, the seedlings from the control and RZT 20°C treatment conditions had 7–8 leaves, with 3–4 of the leaves being emerging new leaves (data not shown). The seedlings treated with RZTs of 5, 10, and 15°C showed minor leaf length and width changes, and only one emerging new leaf, resulting in five leaves at day seven. Younger leaves of 4.0–5.0 cm in length were harvested from the RZT and control conditions, and their morphology analyzed, as illustrated in Figure 1. The visible redness of the leaves increased with an increase in the period of the RZT treatment. However, on the first day of treatment, seedlings treated with RZTs of 5, 10, and 15°C showed leaf wilting. The amaranth leaf fresh and dry weights were 1.05–1.39 g and 0.12–0.21 g, respectively (Figure 2). The RZT treatments at 5 and 10°C had significantly reduced leaf fresh weight when compared with those observed following the treatments at 15 and 20°C. When the treatment period was prolonged from 3 to 7 days, considerable increases in leaf fresh and dry weights were observed; however, 1 day of treatment resulted in a decrease in fresh weight. Notably, extension of the treatment period from 3 to 7 days increased leaf dry weight in the RZT treatments at 15 and 20°C significantly when compared with that observed in the control group (Figure 2). Leaf water content in the control seedlings was consistently between 88 and 90%, while seedlings treated with RZTs of 5, 10, and 15°C exhibited reduced leaf water content since the first day of treatment, when compared with that in the control. However, as the treatment period was prolonged, leaf water content in seedlings treated with RZTs of 5, 10, and 15°C increased gradually.

**Figure 3:**
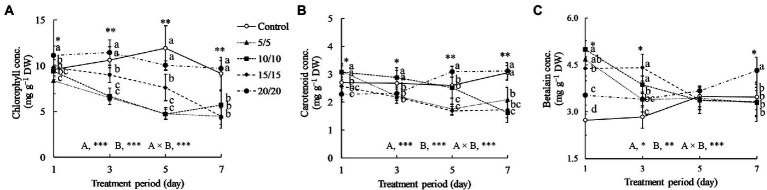
Total chlorophyll **(A)**, carotenoid **(B)**, and betalain **(C)** concentrations (conc.) in leaves of amaranth seedlings treated with different short-term RZTs for different periods (Experiment 1). Results of two-way analysis of variance for RZT (A), period (B), and their interaction (A × B) are shown. The asterisks indicate the significance levels (^*^p < 0.05, ^**^p < 0.01, and ^***^p < 0.001). Vertical bars indicate the standard error (*n* = 4). Means were compared using Tukey’s HSD at a significance level at ^*^p < 0.05 and ^**^p < 0.01. Betalain concentrations are expressed as β-cyanin and β-xanthin equivalents. DW, dry weight.

Figure 3 indicates that the temperatures and periods used for the RZT treatments affected amaranth leaf pigments significantly. When the treatment period was extended from 1 to 7 days, chlorophyll concentrations in the leaves grown at RZTs of 5, 10, and 15°C were 1.25–2.25 times lower than those observed in control and an RZT of 20°C. For one-day treatments, the seedlings grown at RZTs of 5 and 10°C yielded significantly more carotenoids than those grown under control conditions; however, the carotenoid concentrations decreased with a longer treatment period. From 1 to 7 days of treatment, carotenoid concentrations in the leaves grown at an RZT of 20°C increased gradually. Betalain concentrations in the seedlings grown at RZTs of 5, 10, and 15°C increased dramatically 1.2–1.8 times for one-day treatments, and decreased gradually when the treatment period was extended to 7 days.

**Figure 4:**
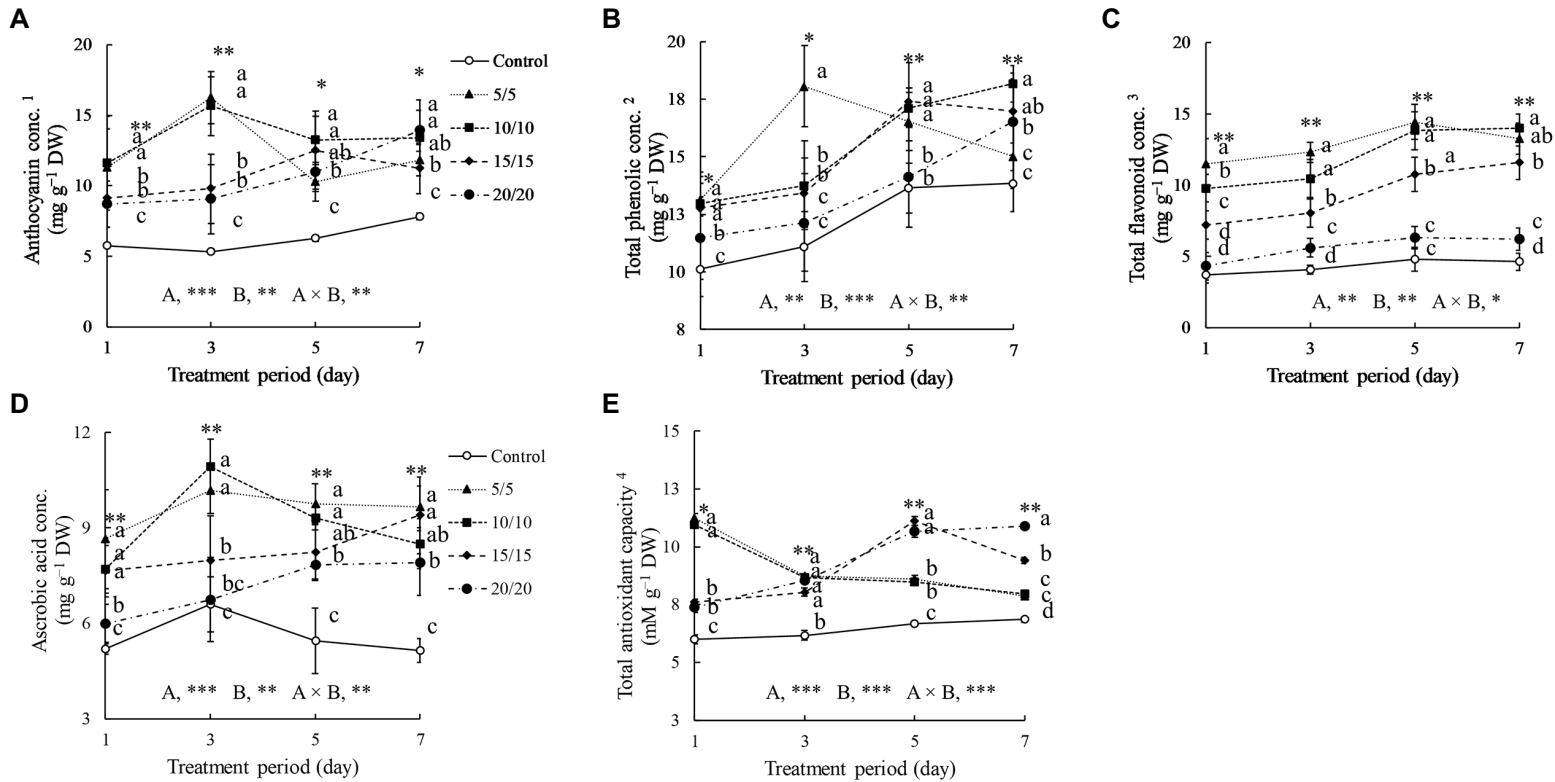
Anthocyanin **(A)**, total phenolic **(B)**, total flavonoid **(C)**, and ascorbic acid **(D)** concentrations, as well as total antioxidant capacity **(E)** of amaranth leaves treated with different short-term root-zone temperatures (RZT) for different periods (Experiment 1). Results of two-way analysis of variance for RZT (A), period (B), and their interaction (A × B) are shown. The asterisks indicate significance levels (^*^p < 0.05, ^**^p < 0.01, and ^***^p < 0.001). Vertical bars indicate the standard error (*n* = 4). Means were compared using Tukey’s HSD at a significance level at ^*^p < 0.05 and ^**^p < 0.01. ^1^Anthocyanin concentration is expressed as cyanidin-3-glucoside equivalents. ^2^Total phenolic concentrations are expressed as gallic acid equivalents. ^3^Total flavonoid concentrations are expressed as rutin equivalents. ^4^Total antioxidant capacity is expressed as Trolox equivalents. DW, dry weight.

The anthocyanin concentrations of seedlings subjected to RZT treatments were 1.3–2.3 times higher than that in the control group (Figure 4). After 3 days of treatment, the anthocyanin concentrations in the seedlings grown at RZTs of 5 and 10°C were increased significantly, up to 16.23 and 15.65 mg g^−1^ DW, respectively. The anthocyanin concentrations of seedlings grown at RZTs of 15 and 20°C increased gradually from day one to day seven of the treatment. Following 3 days of RZT treatment, the total phenolic concentrations in the seedlings grown at n RZT of 5°C increased to 18.06 mg g^−1^ DW, and was the highest among the RZT treatments. However, after 7 days of treatment, total phenolic concentrations in seedlings grown at n RZT of 5°C decreased slightly, whereas the anthocyanin concentrations increased under RZT treatments of 10, 15, and 20°C.

The RZT treatments increased flavonoid concentrations in amaranth leaves significantly during the treatment period, excluding in the RZT of 20°C, in which the was not significant different with that in the control (Figure 4). Extending the treatment periods from 1 to 7 days enhanced leaf flavonoid concentrations, particularly in seedlings grown at RZTs of 5 and 10°C on day 3 to 5. From day 1 to 3, the antioxidant capacity of control seedlings was slightly altered, whereas those of seedlings subjected to RZT treatments were significantly changed. On the first day of treatment, both the 5 and 10°C RZT treatments exhibited the highest antioxidant capacity (11.23 and 10.97 mM g^−1^ DW, respectively), which eventually decreased by 15–20%. Antioxidant capacity increased gradually over 5 days after the start of the RZT treatments at 15 and 20°C.

The concentrations of ascorbic acid were influenced by RZTs and treatment periods (Figure 4). On day three, RZT treatments of 5 and 10°C significantly increased ascorbic acid concentrations in amaranth leaves, whereas the other RZT treatments only marginally increased ascorbic acid concentrations when treatment period was extended.

Exposing amaranth seedlings to RZTs of 5 and 10°C for 1 to 3 days could enhance the contents of bioactive compounds; however, prolonging the treatment period to 7 days reduced the contents of the compounds. From days three to seven, photosynthesis-related pigments and target bioactive compounds increased gradually under an RZT of 20°C. Combining the RZTs could enhance the concentrations of target bioactive compounds and with no leaf abnormalities.

### The establishment of RZTs integration regime

**Figure 5:**
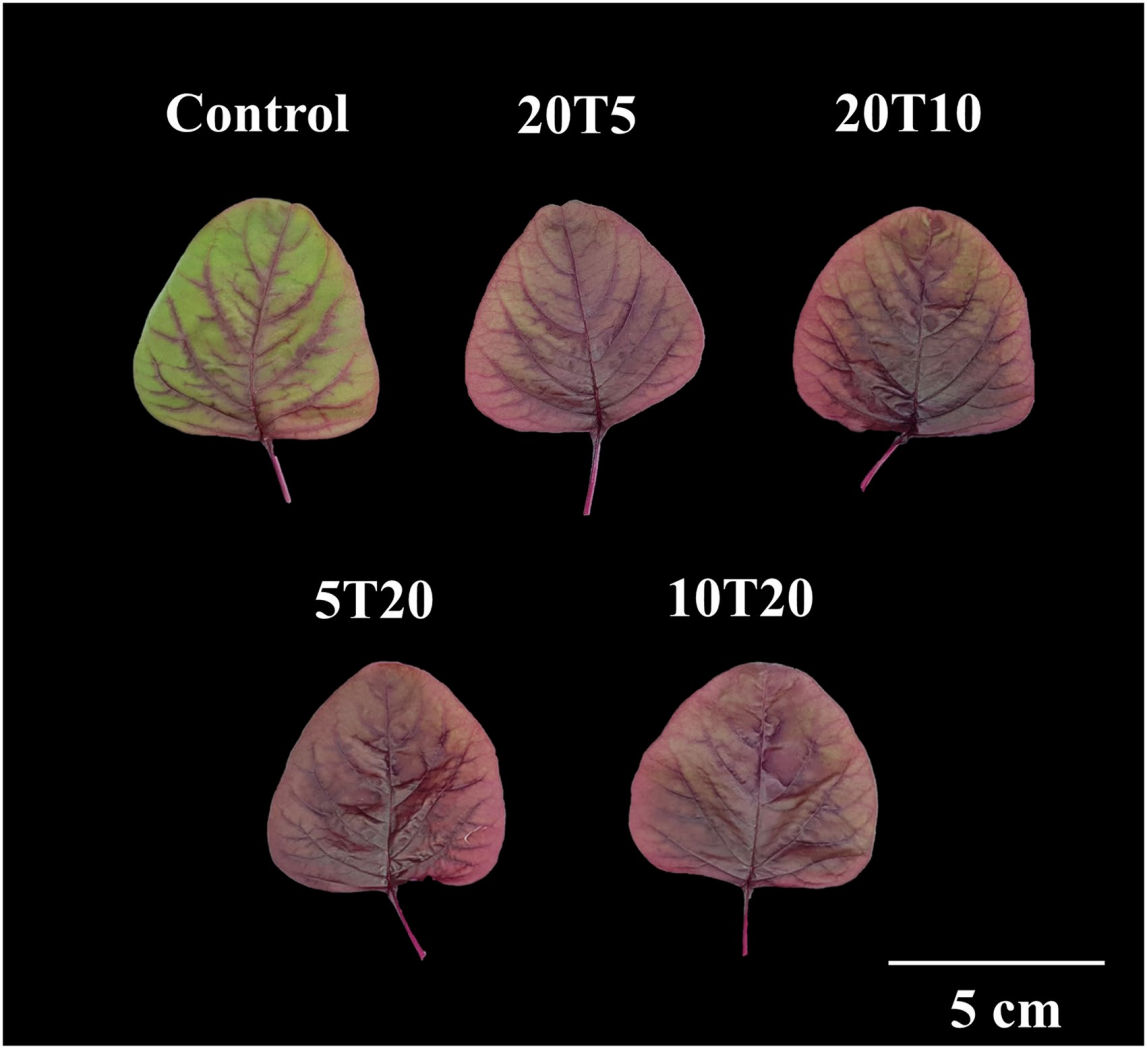
Morphology of amaranth leaves treated with different short-term root-zone temperatures (RZT) treatments for 3  days (Experiment 2). The seedlings that were subjected to 2 days of an RZT of 20°C followed by 1 day of RZTs of 5 or 10°C are designated as 20 T5 and 20 T10, respectively. The seedlings that were treated at 5 or 10°C for 1 day, then subjected to an RZT of 20°C for 2 days, are illustrated as 5 T20 and 10 T20.

The leaf was almost entirely red without wilting symptoms after 3 days of RTZ treatment, whereas the leaves of the 20 T5 and 20 T10 treatments were red and partially green (Figure 5). The leaf fresh weight of RZT-treated seedlings ranged between 1.04 and 1.09 g, which was significantly different from that of the control seedling (1.19 g; Table 3). Leaf dry weight was not significantly different between the RZT-treated seedlings and the control seedlings. In this experiment, the RZT treatment had no significant effect on leaf water content.

**Table 3:** Amaranth leaves fresh weight (Leaf FW), dry weight (Leaf DW), and leaf water content (LWC) after treatment with different short-term root-zone temperatures (RZT) for 3 days (Experiment 2).

Treatment	Leaf FW (g)	Leaf DW (g)	LWC (%)
25/20°C (control)	1.19 ± 0.07^a^	0.19 ± 0.01	84.80 ± 1.23
20 T5	1.06 ± 0.01^b^	0.16 ± 0.02	85.14 ± 0.88
20 T10	1.04 ± 0.02^b^	0.17 ± 0.01	85.25 ± 1.03
5 T20	1.07 ± 0.06^b^	0.16 ± 0.02	85.45 ± 1.18
10 T20	1.09 ± 0.02^b^	0.18 ± 0.02	83.18 ± 1.43
Significant	**	NS	NS

NS indicates no statistical significance.

Data are mean ± standard error of four biological replicates. Means within columns were compared using Tukey’s honestly significant difference (HSD). Different letters indicate significant differences within column. The asterisks indicate the significance level (**p < 0.01).

RZT treatment integration had no effect on photosynthetic pigments, including chlorophyll and carotenoid contents, in the present study (Table 4). The chlorophyll and carotenoid concentrations in the leaves of RZT-treated seedlings ranged from 7.44 to 8.08 and 2.48 to 2.53 mg g^−1^ DW, respectively, which were not significantly different from those of the control. Betalain concentrations in RZT-treated seedlings increased 1.5 to 1.8-times compared to that observed in the control; however, they did not differ significantly among the RZT treatments (3.81–4.21 mg g^−1^ DW).

**Table 4:** Total chlorophyll (Chl conc.), carotenoid (Car conc.), and betalain (Bet conc.) concentrations in leaves of amaranth seedlings after treatment with different short-term root-zone temperatures (RZT) for 3 days (Experiment 2).

Treatment	Chl conc. (mg g^−1^ DW)	Car conc. (mg g^−1^ DW)	Bet conc. (mg g^−1^ DW)
25/20°C (control)	9.71 ± 0.90	2.41 ± 0.15	2.51 ± 0.35^b^
20 T5	7.44 ± 0.83	2.48 ± 0.28	3.95 ± 0.54^a^
20 T10	7.55 ± 1.01	2.49 ± 0.70	3.94 ± 0.56^a^
5 T20	8.08 ± 1.04	2.51 ± 0.49	4.21 ± 0.19^a^
10 T20	7.78 ± 0.97	2.53 ± 0.31	3.81 ± 0.35^a^
Significant	NS	NS	**

Data are mean ± standard error of four biological replicates. Means within columns were compared using Tukey’s HSD. Different letters indicate significant differences within column. The asterisks indicate the significance level (**p < 0.01).

NS indicates no statistical significance. DW, dry weight.

Anthocyanin concentrations in the leaves of RTZ-treated seedlings ranged between 11.15 and 13.15 mg g^−1^ DW, which is approximately 1.3–1.5 times higher than that of the control (Table 5). In addition, RZT-treated seedlings had a phenolic concentration 1.2–1.4 times higher than that of control seedlings.

**Table 5:** Anthocyanin (Ant conc.), total phenolic (Phl conc.), and total flavonoid (Flv conc.) concentrations, as well as total antioxidant capacity (TAC), and ascorbic acid (Asc conc.) concentration of amaranth seedling leaves treated with different short-term root-zone temperatures (RZT) for 3 days (Experiment 2).

Treatment	Ant conc.^1^ (mg g^−1^ DW)	Phl conc.^2^ (mg g^−1^ DW)	Flv conc.^3^ (mg g^−1^ DW)	TAC^4^ (mM g^−1^ DW)	Asc conc. (mg g^−1^ DW)
25/20°C (control)	8.11 ± 0.58^b^	11.53 ± 0.58^c^	4.10 ± 0.21^b^	8.33 ± 0.97^c^	6.78 ± 0.40^c^
20 T5	13.15 ± 1.15^a^	15.49 ± 1.13^ab^	13.13 ± 0.93^a^	13.45 ± 0.95^ab^	9.90 ± 0.38^b^
20 T10	13.14 ± 1.58^a^	16.68 ± 0.80^a^	12.00 ± 0.99^a^	15.51 ± 1.22^a^	11.05 ± 0.71^a^
5 T20	12.61 ± 0.87^a^	16.34 ± 0.83^a^	12.45 ± 1.22^a^	14.09 ± 1.08^ab^	11.70 ± 0.84^a^
10 T20	11.15 ± 1.04^a^	14.40 ± 0.83^b^	11.06 ± 0.94^a^	11.85 ± 0.57^b^	9.55 ± 0.51^b^
Significant	**	**	**	**	**

1 Anthocyanin concentrations are expressed as cyanidin-3-glucoside equivalents.

2 Total phenolic concentrations are expressed as gallic acid equivalents.

3 Total flavonoid concentrations are expressed as rutin equivalents.

4 Total antioxidant capacity is expressed as Trolox equivalents.

Data are mean ± standard error of four biological replicates. Means within columns were compared using Tukey’s HSD. Different letters indicate significant differences within column. The asterisks indicate the significance level (**p < 0.01). NS indicates no statistical significance. DW, dry weight.

Integration of RZT treatments increased flavonoid concentrations significantly (2.7–3.0 times higher than that in the control). There were no significant differences in flavonoid concentrations among the RZT treatments. The highest phenolic concentrations (16.68 mg g^−1^ DW) were observed in leaves in the RZT 20 T10 treatment, whereas the lowest were observed in leaves in the RZT 10 T20 treatment. The findings are consistent with the antioxidant capacity of RZT-treated leaves, which was the highest in the 20 T10 treatment (15.51 mM g^−1^ DW) and the lowest in the 10 T20 treatment (11.85 mM g^−1^ DW), among the RZT treatments. Both phenolic concentration and antioxidant capacity of seedlings were significantly different between the RZT 20 T10 and RZT 10 T20 treatments. Interestingly, RZT 20 T10 and RZT 10 T20 protocols were designed using the same RZTs of 10 and 20°C, but different periods, implying that a different RZT introduction time at 10°C results in different phenolic concentration and antioxidant capacity. Conversely, a different RZT introduction time at 5°C showed a similar result.

The effects of RZTs integration treatments on ascorbic acid are listed in Table 5. The results indicate that both the 5 and 10°C RZTs can be used to increase ascorbic acid concentrations; however, the introduction period influenced the results considerably. RZT should be applied for 1 day at 5°C, followed by 2 days at 20°C (5 T20), whereas 10°C for 1 day should be used after seedlings have been treated for 2 days at 20°C (20 T10).

The results of the present study suggest that RZT integration could enhance the contents of target bioactive compounds. The application of an RZT of 5°C for one day, followed by an RZT of 20°C for two days (5 T20), represents a promising protocol for improving bioactive compound accumulation in baby leaf amaranth without inducing abnormal appearance.

## Discussion

Numerous studies on RZT have examined its influence on hydroponically grown plants, with an emphasis on low RZTs. Low RZTs have been associated with adverse effects, such as reduced water and nutrient uptake, decreased root respiration and activity, retarded root development, or interruptions of the photosynthetic machinery, all of which result in loss in product yield, nutrients, and minerals (Aroca et al., 2001; Schwarz et al., 2010; Hao et al., 2012). Alternatively, low RZTs are considered beneficial since short- or long-term application can enhance the concentrations of bioactive compounds, such as rosmarinic acid (Lam et al., 2020), glucosinolates (He et al., 2020), ascorbic acid (Chadirin et al., 2011), total phenolics, and anthocyanin (Sakamoto and Suzuki, 2015), depending on the plant species.

### Growth and photosynthesis-related pigments

Plant biomass is a growth indicator that reflects photosynthetic status and stress, so that a decrease in plant biomass implies poor growth or excessive stress. This study showed that the seedlings grown at RZTs of 5 and 10°C had a substantial decrease in fresh weight (Figure 2). Moreover, one-day treatments resulted in significant reduction in fresh weight. Sakamoto and Suzuki (2015) suggested that treating red leaf lettuce using an RZT of 10°C for 7 days noticeably decreased the fresh weight. According to Nguyen et al. (2020), although the fresh weight of coriander exposed to a 15°C RZT treatment did not change significantly after three days, it did after six days. The RZTs used and the treatment periods influenced plant fresh weight by inducing an imbalance in root water uptake. Li et al. (2019b) demonstrated that rice plants grown at an RZT of 17.5°C for two days increased malonaldehyde concentrations, reflecting oxidative status in the root and shoot, and decreasing shoot fresh and dry weights. Low RZT potentially disturbed redox balance, generating an overproduction of ROS that necessitated the synthesis of neutralizing antioxidant enzymes or bioactive compounds (Escobar-Bravo et al., 2017). Such activities require energy to be performed; hence, respiration rate is increased. Due to the impairment of root water uptake, water is lost throughout respiration without being replenished by the root, resulting in loss of shoot fresh weight (Calleja-Cabrera et al., 2020). However, in the present study, the extended RZT treatment period increased leaf dry weight gradually, with a decrease observed only at the first day of treatment. The RZT treatments applied in the present study were potentially suboptimal and did not exceed amaranth’s biological threshold, allowing the recovery process to proceed.

LWC is a parameter used to measure the leaf water stress status in response to drought, temperature, and nutrient limitation (Heinen et al., 2009). On the first day of treatment, RZT leaves exposed to 5, 10, and 15°C showed significant decreases in LWC (Figure 2). Therefore, the one-day RZT treatment may not be appropriate for use prior to harvest. However, extension of RZT period increased LWC significantly, while seedlings grown in the control and under an RZT of 20°C exhibited minor changes in LWC (Figure 2). Various physiological responses are triggered, in general, to maintain water levels in plants during water stress, including alteration of stomata conductance (Tari, 2003; Yu et al., 2015) proline biosynthesis (Sperdouli and Moustakas, 2014; Zegaoui et al., 2017), and the accumulation of photosynthesis-related pigments (Sanchez et al., 1983; Mibei et al., 2017; Shah et al., 2017).

Decreases in chlorophyll concentrations in seedlings grown under RZTs of 5, 10, and 15°C may indicate poor light-harvesting ability for photosynthesis (Figure 3). Low RZT treatments cause lipid oxidation in the membrane, resulting in ROS production, most notably in the plastid. This ROS subsequently oxidizes the pigment, decreasing the chlorophyll content (León-Chan et al., 2017). The decrease may influence plant biomass, as previously described, by lowering seedling fresh and dry weights under low RZT treatments. The negative trends, especially for fresh weight, are more obvious when the treatment period is prolonged.

Carotenoids, in general, are synthesized in the same way as chlorophyll, highlighting their role in the photosynthetic machinery (Sagawa et al., 2016; Stanley and Yuan, 2019). Carotenoids are pigments accumulated in chloroplasts and crucial for photoprotection, capturing light, and stabilizing photosynthetic activities (Havaux, 1998), and have a high antioxidant capability, scavenging singlet oxygen and peroxyl radicals (Stahl and Sies, 2003). In the present study, carotenoid concentrations increased significantly in seedlings exposed to RZTs of 5 and 10°C on the first day but decreased thereafter, by 30–40%, when the treatment period was extended. Under cold temperatures, the elongated hypocotyl 5 (HY5), a protein that responds to cold and light stress, stabilized; however, the phytochrome interacting factor decreased antagonistically (Toledo-Ortiz et al., 2014). Both proteins interacted with the phytoene synthase enzyme associated with carotenoid biosynthesis under cold stress. However, plant age, leaf position, and phytohormone levels influence carotenoid stability. Low RZTs suppress phytohormones such as gibberellins, auxin, and cytokinin in root apical meristems, in turn interfering with root-to-shoot hormonal transportation (Zhu et al., 2015; Müller and Munné-Bosch, 2021). Consequently, in amaranth seedlings, low RZT treatments lasting longer than 3 days may restrict the biosynthesis of photosynthesis-related pigments and reduce leaf growth.

### Bioactive compounds

Betalain, a tyrosine-derived pigment, is required for homeostasis under abiotic stress. Betalain accumulates in the epidermis, mesophyll, and guard cell of leaves (Jain and Gould, 2015; Zhou et al., 2021). In amaranth leaves, especially red leaves, betalain concentrations are as much as 2–5 times higher than those in green leaves (Liu et al., 2019). Hayakawa and Agarie (2010) reported that cold stress enhanced betalain concentrations in *Suaeda japonica* leaves. Numerous cold-responsive bioactive compounds are synthesized to maintain photosynthetic machinery under cold stress. Betalain, rather than carotenoid, is a photoprotective compound strongly associated with cold stress (Li et al., 2019a). Betalain concentrations increased significantly in leaves under RZT treatments at 5, 10, and 15°C, and slightly reduced (Figure 3). In general, the biosynthesis of betalain requires nitrogen as a backbone, similar to chlorophyll, potentially explaining the observed decrease in chlorophyll and increase in betalain under RZT treatments. Jain and Gould (2015) showed that betalain concentrations increased 4-fold when exposed to water stress, while chlorophyll concentrations decreased dramatically. Under low RZT conditions, photosynthesis is restricted, which leads to the depletion of plant resources in tissues and forces them to preserve critical nutrients for survival. Chlorophyll may be less necessary in a resource-limited environment with excess ROS when compared with betalain and carotenoid (antioxidants; Polturak et al., 2016).

Anthocyanins have been commonly identified as plant stress-responsive bioactive molecules, acting as ROS scavengers and osmo-homeostatic agents (Stintzing and Carle, 2004). Numerous studies have showed that RZT treatments increase anthocyanins in leaves significantly. For example, anthocyanin concentrations increased in red leaf lettuce after exposure to low RZTs of 10 and 15°C for a certain period (Sakamoto and Suzuki, 2015). Similarly, the leaves of red perilla grown at an RZT of 10°C for 6 days exhibited a considerable increase in anthocyanin concentrations when compared with that in the control (Ogawa et al., 2018). In the present study, anthocyanin concentrations in amaranth leaves under RZT treatments responded variably depending on the RZT and treatment period (Figure 4). For instance, anthocyanin concentration increased dramatically, 2–3-fold, after treatment at 5 and 10°C for 3 days, when compared with that observed in the control, followed by a 15–20% decline on days 5 and 7. The leaves under 15 and 20°C treatments progressively produced anthocyanins as the treatment periods were extended, indicating that RZT treatments of 5 and 10°C may be the low threshold temperature level of amaranth, triggering the biosynthesis of anthocyanin components by activating chalcone synthase, chalcone isomerase, and flavanone-3-hydroxylase (Liu et al., 2018). However, the reduction in anthocyanin, known as anthocyanin degradation, may begin after day 3 of RZT treatment. During RZT treatment, photosynthesis is limited, with sugar lacking as an energy source, and plants are forced to recycle sugar to survive. Anthocyanins in general, which are present as glycosides, bonded with a sugar-backbone, may be targeted as alternative sugar sources. Therefore, the deglycosylation by β-glucosidase and oxidation by polyphenol oxidase or peroxidase may potentially result in anthocyanin degradation (Oren-Shamir, 2009). Anthocyanin performs its ROS scavenging function in a manner similar to betalain. Both bioactive compounds may be used initially during the RZT treatment; afterward, additional stress-responsive components will continue to perform the function. In the present study, anthocyanin and betalain reduction were observed on day 5 and 7 (Figures 3, 4).

Król et al. (2015) demonstrated that after a week of exposure to 10°C, total phenolic contents of *Vitis vinifera* L. leaves increased. However, the cold-sensitive cultivar had lower phenolic contents. The data suggest that the phenolic compounds play a role in cold tolerance. After 30 days of long-term low-temperature stress at 15°C, the accumulation of flavonoids and phenolic compounds in tomato leaves increased (Rivero et al., 2001). Rezaie et al. (2020) demonstrated that low RZTs induce transcription factors associated with phenylalanine ammonia-lyase and alter the expression of the cinnamate 4-hydroxylase gene, resulting in increased flavonoid and phenolic compound accumulation in sweet basil, which corroborate the findings of the present study, revealing that seedlings exposed to an RZT of 5°C for 3 days significantly enhanced their total phenolic concentration (Figure 4). The findings indicate that seedling leaves treated with different RZTs (5, 10, and 15°C) exhibited 3–5-fold increases in flavonoid concentrations when compared with those of the controls. When the treatment period was prolonged, total phenolic and flavonoid concentrations increased substantially, showing that both are required for homeostasis under low RZTs. Cold-tolerance capacity varies according to plant species, based on cold-responsive transcriptional factors. Moreover, different mechanisms may be adopted depending on the magnitude and period of the cold stress.

Lee and Oh (2015) reported that the antioxidant capacity of kale treated with an RZT of 4°C increased significantly on the first day after treatment. Consequently, the antioxidant capacity of leaves exposed to RZTs of 5 and 10°C increased dramatically by 1.5–1.8-fold, when compared with those observed after 1 day of treatment in the control (Figure 4). The antioxidant capacity of seedling leaves treated at 15 and 20°C increased gradually from day one to seven. Plant ROS defense mechanisms are diverse and include both enzymatic and non-enzymatic responses. Both are employed considering the quantity of ROS and the physiological status of the plant (Das and Roychoudhury, 2014). The antioxidant capacity of amaranth is strongly correlated with the concentration of bioactive compounds classified as non-enzymatic antioxidants, such as anthocyanin, betalain, phenolic, flavonoids, and ascorbic acid (Sarker et al., 2020). However, only betalain and carotenoid increased with an increase in antioxidant capacity, whereas phenolic, flavonoids, and ascorbic acid exhibited delayed responses (Figures 3, 4). Carotenoid and betalain antioxidant molecules may be the first line of defense against ROS damage in amaranth during RZT treatment. However, based on reactivity and structure, phenolic and flavonoid compounds are considered secondary ROS scavenging mechanisms in plants (Fini et al., 2011). Additionally, delayed synthesis resulting from delayed gene expression in response to abiotic stress has been described in other studies (Clayton et al., 2018; Qian et al., 2019).

Ascorbic acid is a water-soluble vitamin that is essential for the immune system and as a cellular ROS scavenger. Moreover, it is more effective in scavenging hydrogen peroxide than catalase and peroxidase (Dolatabadian and Jouneghani, 2009). Amaranth is a source of ascorbic acid, particularly from its leaves, containing 13 times the ascorbic acid content found in lettuce (Srivastava, 2011). In the present study, ascorbic acid increased significantly in leaves treated at 5°C from day one and remained constant until day seven, but it decreased slightly in leaves treated at 10°C after 7 days (Figure 4). Ascorbic acid functions as a scavenger in both enzymatic and non-enzymatic ROS defense mechanisms, implying that ascorbic acid biosynthesis may continue under abiotic stress (Barnes et al., 2002; Akram et al., 2017). Here, we showed that ascorbic acid increased in seedlings subjected to RZT treatments at 5 and 10°C for 3 days, and declined slightly thereafter. Subsequently, Ito and Shimizu (2020) observed that spinach (Amaranthaceae) exposed to 4°C treatment for 2–7 days exhibited enhanced levels of ascorbic acid. Moreover, Cheminant et al. (2011) reported that after 2 weeks of RZT treatment at 5°C, the ascorbic acid content in spinach increased. Although previous studies examined the influence of RZT treatments on ascorbic acid in spinach, the effects may differ based on the species analyzed. Ascorbic acid is produced from glucose, pectin, and myo-inositol; hence, higher ascorbic acid levels indicate increased utilization of the substrates (Wheeler et al., 1998; Smirnoff et al., 2001). Since these precursors influence plant growth and development, long-term RZT treatment of amaranth may not be appropriate, as demonstrated in the present study.

### Effect of cooling root-zone temperature integration

Since each RZT level and period (Experiment 1) had distinct effects, an integration of varied RZTs for 3 days before harvest may be performed to enhance the concentrations of bioactive compounds. An RZT of 20°C may increase photosynthetic pigments, providing precursors of bioactive compounds and nutrients; hence, 2 days would be selected for this RZT. After the 20°C treatment, RZTs of 5 or 10°C would be used for a day to improve the bioactive compounds and nutrients before harvest. Conversely, RZTs of 5 and 10°C may be first used for a day to enhance the concentrations bioactive compounds and nutrients, followed by an RZT of 20°C to gradually increase and maintain them until harvest.

While cooling the root zone can enhance the concentrations of bioactive compounds (Ogawa et al., 2018; Nguyen et al., 2020), it also affects leaf fresh weight, in general, by interfering with root water uptake (Nxawe et al., 2009; Maurel et al., 2015). According to the results presented in Figure 2, the RZT of 20°C had no effect on leaf fresh weight when compared with that of the control group, and hence was employed in Experiment 2 to avoid leaf fresh weight reduction. However, the conclusion may not be valid, since the results indicated that seedlings treated with a combination of RZTs exhibited leaf fresh weight reductions of 10–15% compared to the control (Table 3). Notably, the combination of RZTs was observed to have a beneficial effect on plants by reducing water stress damage, as the dry weight and leaf water content indicated a water and nutrient balance (Calleja-Cabrera et al., 2020; Zhou et al., 2021), which were not statistically different from those of the control (Table 3).

In experiment 1, chlorophyll may have been degraded by lipid oxidation (León-Chan et al., 2017) and reduced to a basal level for the maintenance of physiological processes under resource-constrained conditions induced by low RZTs (Figure 3). Notably, the findings in Experiment 2 suggest that the combination of RZTs had a beneficial effect on chlorophyll, despite the no significant difference between the RZT treatment and the control (Table 4). Chlorophyll may accumulate more when plants are under mild RZT stress, as shown in our experiment with an RZT of 20°C (Figure 3). Moreover, no reduction in chlorophyll concentration was observed when RZTs of 5 and 10°C were introduced (20 T5 and 20 T10, Table 4). The mild cooling RZTs of 15 to 20°C at 25/20°C air temperatures may promote chlorophyll accumulation, thereby increasing photosynthesis and ultimately bioactive compound concentrations or precursor biosynthesis (He et al., 2020). Therefore, when seedlings are exposed to low RZTs, the precursors are promptly converted to cooled RZT-responsive compounds, and the resulting bioactive chemical compounds immediately protect chlorophyll from lipid oxidation. Conversely, when RZTs of 5 and 10°C were introduced prior to the application of 20°C (5 T20 and 10 T20), the 20°C may serve as a recovery condition; various bioactive compounds increased during the recovery period (Lee and Oh, 2015).

RZT combinations had different effects on carotenoids, when compared to the effects observed during Experiment 1, where only a single RZT treatment was applied. The 5 and 10°C RZT treatments in Experiment 1 increased carotenoid concentrations for 1 day, and then decreased them over time (Figure 3); however, there was no significant difference between the RZT treatments and the control (Table 4). Carotenoids, as mentioned earlier, acts as ROS scavengers and photoprotective compounds (Stahl and Sies, 2003); cooling RZTs, in general, lead to increased carotenoid concentrations (Chadirin et al., 2011; Nguyen et al., 2020; He et al., 2021). A cooling RZT activates HY5, a switch for carotenoid biosynthesis (Catalá et al., 2011). However, the synthesis of several bioactive compounds, including flavonoids, is controlled by this protein (Kim et al., 2017). According to the results of the present study, RZT treatments may induce HY5, but carotenoids may not be the primary RZT stress-responsive compound.

In the present study, betalain, anthocyanins, phenolics, and flavonoids functioned as bioactive compounds in response to cooling RZTs (Table 5 and Figures 3, 4). RZTs, either single or in combination, improve these concentrations for a certain period when compared with the concentrations observed in control treatments. However, phenolic concentrations are affected by introduction of RZT treatments at 5 and 10°C followed by or before treatment at 20°C. Among RZT treatments, in the 10 T20 protocol, leaves had the lowest concentrations of phenolic compounds, and their antioxidant capacity was consequently reduced due to the lower phenolic concentrations (Table 5). The antioxidant capacity of amaranth leaves, on the other hand, demonstrates the total ROS scavenging ability (scavenging, neutralizing, and radical reaction chain inhibition; Rubio et al., 2016), which is highly correlated with betalain, phenolic, and flavonoid contents (Sarker and Oba, 2018, 2020).

The integration of RZTs had distinct effects on ascorbic acid contents in leaves (Table 5). Ascorbic acid concentration of seedlings under 20 T5 treatment was lower than that under the 20 T10. In comparison with other bioactive compounds examined following RZT combination treatments, only ascorbic acid concentrations were significantly decreased in leaves after 20 T5, indicating that the RZT of 5°C 1 day prior to harvest may induce cold stress and produce ROS, for which ascorbic acid is required as a first-line scavenger, resulting in the reduction in the ROS concentrations (Akram et al., 2017). However, leaves treated with 10 T20 showed reduced ascorbic acid concentrations, which is associated with decreased phenolic concentrations and antioxidant capacity. In the 10 T20 treatment, an RZT of 10°C induced cold-stress and generated ROS; however, the recovery period at 20°C could have masked the impact of stress, resulting in lower phenolic and ascorbic acid concentrations and antioxidant capacity than those in other combinations.

Pre-cooling, in general, is the application of 10–20°C of forced air to leafy vegetables to remove excess heat from the environment or metabolic process (Martínez and Artés, 1999). Numerous studies have shown that pre-cooling vegetables extends their shelf life by increasing the concentrations of bioactive compounds that function as radical scavengers and chelating agents (He et al., 2013; Garrido et al., 2015; Tian et al., 2016; Kongwong et al., 2019). Therefore, pre-cooling RZTs (20°C in this experiment), in contrast to the cold-RZT warning signal, may enhance bioactive compounds prior to the application of cold-RZTs (5 and 10°C). Alternatively, an RZT treatment of 20°C could be used after applying cold-RZTs (5 and 10°C; 5 T20 and 10 T20) where the RZT of 20°C may not act as a pre-cooling RZT but rather as a recovery-period RZT.

Our findings suggest that a one-day RZT treatment combination at 5°C followed by two-days treatment before harvest at 20°C can enhance the concentrations of target bioactive compounds and maintenance of nutrients in baby leaf amaranth without adversely affecting leaf appearance. Amaranth baby leaf responded differently to different RZT combinations, and this is the first study to report such findings. Further studies are required to demonstrate the relationship between pre-harvest treatment and post-harvest quality of baby leaf amaranth since physiological processes in plants change over time, even after harvest.

## Conclusion

Our findings suggest that RZTs of 5 and 10°C for 1–3 days may increase the concentrations of bioactive compounds in baby leaf amaranth, with beneficial nutrients retained. An RZT of 20°C, conversely, would increase the concentrations of photosynthetic pigments and bioactive compounds without impairing growth, when the treatment period is extended to 7 days. Furthermore, the combination of RZTs could enhance target bioactive components and maintain nutrients in amaranth baby leaf. Treatments for 1 day at 5°C and for 2 days at 20°C were associated with the highest concentrations of bioactive compounds and nutrients in the leaves. Therefore, treatment with an RZT of 5°C for 1 day followed by that for 20°C for 2 days may be the appropriate pre-harvest treatment for increasing bioactive compounds and maintaining nutrients in baby leaf amaranth without causing leaf appearance abnormalities.

## Data availability statement

The original contributions presented in the study are included in the article/Supplementary material, further inquiries can be directed to the corresponding author.

## Author contributions

EG and KS: conceptualization and supervision. TW: performing experiments. EG, KS, and PW: chemical analysis advice. TW: growth parameters and biochemical analysis, data analysis, and writing of manuscript. EG: review and editing of manuscript and funding acquisition. All authors contributed to the article and approved the submitted version.

## Funding

This work was supported by JSPS KAKENHI Grant Number JP18H02301 and a Thailand Graduate Institute of Science and Technology (TGIST) Scholarship (SCA-CO-2560-4576-TH).

## Conflict of interest

The authors declare that the research was conducted in the absence of any commercial or financial relationships that could be construed as a potential conflict of interest.

## Publisher’s note

All claims expressed in this article are solely those of the authors and do not necessarily represent those of their affiliated organizations, or those of the publisher, the editors and the reviewers. Any product that may be evaluated in this article, or claim that may be made by its manufacturer, is not guaranteed or endorsed by the publisher.

## Supplementary materials

The Supplementary materials for this article can be found online at: https://www.frontiersin.org/articles/10.3389/fpls.2022.944716/full#supplementary-material
