# Supplementary material for: A short-term cooling of root-zone temperature increases bioactive compounds in baby leaf Amaranthus tricolor L
Source: Front Plant Sci. 2022 Jul 15;13:944716. doi: 10.3389/fpls.2022.944716 (PMC9335047; doi:10.3389/fpls.2022.944716)
Supplement: Supplementary file 1 [file Data_Sheet_1.docx]

Supplementary Material

**Table S1.** Air temperature (AT), canopy temperature (CT), leaf surface temperature (LST), expanded polystyrene cultivation foam surface temperature (EPST), air temperature under cultivation foam (ATU) and nutrient solution temperature (NST) of RZT treatments.

| Treatment | Period |  | | Temperature (°C) | | | | |
| --- | --- | --- | --- | --- | --- | --- | --- | --- |
|  |  | AT^1^ | CT | | LST | EPST | ATU | NST |
| Control | Light | 24.82 ± 0.33 | 25.64 ± 0.39 | | 25.44 ± 0.57 | 25.10 ± 0.46^a^ | 24.19 ± 0.42^a^ | 24.69 ± 0.62^a^ |
| 5/5°C |  | 24.95 ± 0.34 | 25.59 ± 0.29 | | 25.06 ± 0.54 | 22.65 ± 0.67^b^ | 10.75 ± 0.41^e^ | 5.01 ± 0.51^e^ |
| 10/10°C |  | 24.95 ± 0.34 | 25.70 ± 0.39 | | 25.25 ± 0.58 | 23.90 ± 0.57^ab^ | 13.88 ± 0.52^d^ | 10.03 ± 0.57^d^ |
| 15/15°C |  | 24.89 ± 0.38 | 25.97 ± 0.52 | | 25.13 ± 0.76 | 24.20 ± 0.71^a^ | 16.95 ± 0.39^c^ | 15.78 ± 1.09^c^ |
| 20/20°C |  | 24.89 ± 0.38 | 25.85 ± 0.35 | | 25.00 ± 0.71 | 24.80 ± 0.59^a^ | 20.67 ± 0.43^b^ | 20.00 ± 0.61^b^ |
| Significant | | NS | NS | | NS | ** | ** | ** |
| Control | Dark | 19.34 ± 0.47 | 19.28 ± 0.43^a^ | | 19.25 ± 0.77 | 19.85 ± 0.47^a^ | 19.45 ± 0.29^a^ | 19.83 ± 0.53^a^ |
| 5/5°C |  | 19.33 ± 0.48 | 18.90 ± 0.46^b^ | | 19.18 ± 0.66 | 17.75 ± 0.49^b^ | 9.04 ± 0.26^d^ | 4.83 ± 0.26^d^ |
| 10/10°C |  | 19.33 ± 0.48 | 19.08 ± 0.44^a^ | | 19.31 ± 0.48 | 19.65 ± 0.34^a^ | 12.48 ± 0.48^c^ | 9.62 ± 0.45^c^ |
| 15/15°C |  | 19.35 ± 0.49 | 19.18 ± 0.50^a^ | | 19.53 ± 0.46 | 19.55 ± 0.64^a^ | 15.79 ± 0.43^b^ | 15.63 ± 1.03^b^ |
| 20/20°C |  | 19.35 ± 0.49 | 19.32 ± 0.50^a^ | | 19.31 ± 0.63 | 19.80 ± 0.48^a^ | 18.70 ± 0.42^a^ | 19.84 ± 0.46^a^ |
| Significant | | NS | * | | NS | ** | ** | ** |

Data are mean ± standard deviation. The AT, CT, and ATU were recorded every minute for one day, from 8:00 to 21:59 for the light period (n = 3) and 00:00 to 6:59 for the dark period (n = 3). The LST of individual leaves (n = 16) and the EPST (n = 10) were determined at 15:00 and 00:00, for light and dark periods, respectively. NST was measured during the light period (n = 3) at 15:00, 19:00, and 22:00 and during the dark period (n = 3) at 23:00, 00:00, and 1:00. NS indicates no statistical significance. ^1^ The RZT treatment of 5/5 and 10/10°C, as well as 15/15 and 20/20°C were coupled and conducted on the same shelf; therefore, AT of those were measured at the same time. Means within columns of each period were compared using Tukey’s HSD. The asterisks indicate significance levels (**p* < 0.05; ***p* < 0.01).


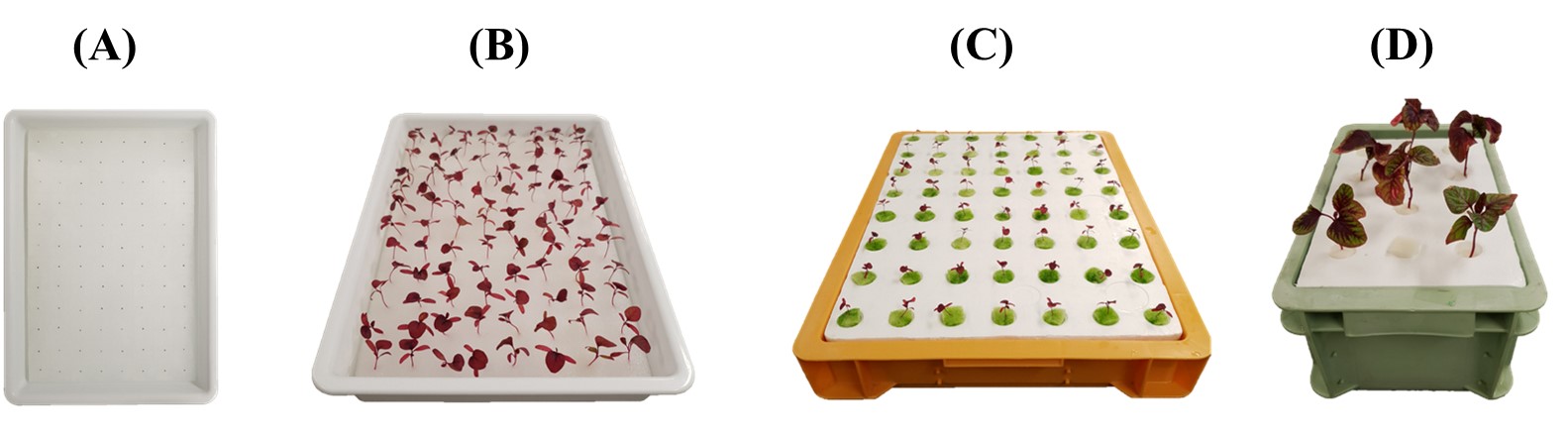


**Figure S1**. Baby-leaf amaranth cultivation procedure includes sowing (A), seedling production (B), seedling spacing (C), and seedling selection for the treatment (D).

**Figure S2**. The appearance diagram of RZT experiments on the cultivation shelf in a closed plant production system.

**Figure S3**. The experimental apparatus for RZT treatment on the cultivation shelf in a close plant production system. White arrows indicate components in the apparatus. 1. Cooling coil. 2. Air supplying tube. 3. Thermocouple with the connected line to a temperature control device. 4. Temperature control device. 5. Handy cooler. 6. Air pump. 7. Air stone.

**Figure S4**. The setting value of root-zone temperature and air temperature (AT) during the treatment~~s~~. The RZTs integration of 20ºC for two days, followed by 5 or 10ºC for one day, represented by 20T5 and 20T10, respectively. The RZTs integration of 5 or 10ºC for one day, followed by 20ºC for two days, represented by 5T20 and 10T20, respectively. The air temperature was 25/20ºC for the light/dark period indicated by white/black boxes.
